# Acalypha australis L. induces autophagic cell death in colorectal cancer cells by increasing ROS through suppression of the AKT/mTOR/HIF-1 pathway

**DOI:** 10.3389/fonc.2026.1795876

**Published:** 2026-03-31

**Authors:** Changpu Du, Jian Li, Kangmin Zhou, Huazheng Sun, Ailing Liang, Rong Tan, Jiancheng Zhai, Wenqi Huang, Bing Yang, Dongxin Tang

**Affiliations:** 1 Guizhou University of Traditional Chinese Medicine, Guiyang, Guizhou, China; 2 The First College of Clinical Medicine, Guizhou University of Traditional Chinese Medicine, Guiyang, Guizhou, China; 3 The First Affiliated Hospital of Guizhou University of Traditional Chinese Medicine, Guiyang, Guizhou, China

**Keywords:** Acalypha australis, autophagy, colorectal cancer, Miao medicine, ROS

## Abstract

**Background:**

Previous studies indicate that Acalypha australis L. (AAL), a traditional Miao medicine, effectively treats colitis, but its anti-tumor effects on colorectal cancer (CRC) are unclear.

**Objective:**

This study aims to investigate the anticancer effects of ethanol extracts of Acalypha australis L. (EEAL) against CRC and elucidate their potential mechanisms of action.

**Methods:**

This study first employed ultra-high-performance liquid chromatography-tandem mass spectrometry (UHPLC-MS/MS) to analyze the chemical components of EEAL and utilized databases like TCMSP to identify potential anticancer compounds against CRC. Network pharmacology was then used to predict the mechanisms and targets of EEAL in CRC treatment. The effects of EEAL on the proliferation of LOVO and HCT116 CRC cells were assessed using CCK-8 assays and colony formation experiments. Changes in membrane integrity were measured with CM-Dil staining, along with mitochondrial membrane potential and intracellular reactive oxygen species (ROS) levels. The extent of autophagy after treatment was evaluated with transmission electron microscopy and Lyso-Tracker staining to observe autophagosome (ASS) expression. Finally, Western blot (WB) analysis was performed to assess the expression of relevant proteins and investigate the potential anticancer mechanisms of EEAL against CRC.

**Results:**

A total of 30 candidate components with potential anticancer activity against CRC were identified from EEAL. Network pharmacology analysis suggested that EEAL may regulate oxidative stress in CRC treatment through the AKT/mTOR/HIF-1 pathway. Cellular experiments demonstrated that EEAL significantly inhibited the proliferation and colony formation ability of LOVO and HCT116 CRC cells. Further studies revealed that EEAL not only decreased mitochondrial membrane potential and increased intracellular ROS levels but also induced significant accumulation of autophagic lysosomes and compromised membrane integrity. Western blot analysis showed that EEAL decreased the protein expression of AKT, P-mTOR, and HIF-1, while upregulating the expression of the autophagy marker LC3B.

**Conclusion:**

This study demonstrates that EEAL exerts its anticancer effects against CRC by inhibiting the AKT/mTOR/HIF-1 pathway, leading to oxidative stress and mitochondrial dysfunction, which subsequently triggers excessive autophagy and compromises cell membrane integrity.

## 1 Introduction

Colorectal cancer (CRC) is the third most commonly diagnosed malignancy worldwide. In 2022, it was estimated that there were over 1.9 million new CRC cases and approximately 904,000 CRC-related deaths globally, accounting for nearly one-tenth of all cancer incidence and mortality. Moreover, the incidence of CRC has been steadily increasing in recent years (1) Despite ongoing advances in surgical techniques, chemotherapy, targeted therapy (2), and immunotherapy (3) that have improved the overall survival rates for CRC, the prognosis for patients with advanced CRC remains poor. Issues such as drug resistance (4), severe toxic side effects (5), and tumor recurrence (6) significantly compromise treatment outcomes. Therefore, there is an urgent need to develop more effective and safer novel antitumor strategies.

In recent years, active ingredients derived from natural products have attracted increasing attention in cancer research due to their multi-targeted effects and relatively low toxicity. Acalypha australis L. (AAL), a representative traditional Miao medicinal herb, is traditionally utilized for treating inflammatory bowel diseases such as colitis due to its notable anti-inflammatory properties (7). Recent pharmacological studies have further validated its therapeutic efficacy in gastrointestinal disorders; for instance, proteomic analysis has revealed that AAL mitigates chronic colitis by preserving intestinal barrier integrity and modulating the FABP4/PPARγ/NF-κB signaling pathway (8). Furthermore, AAL extract has been shown to attenuate DSS-induced ulcerative colitis in mice by reducing the release of pro-inflammatory factors and blocking NF-κB activation, which subsequently decreases colonic apoptosis (9). Given that chronic intestinal inflammation is a critical risk factor for CRC and often serves as a precancerous condition, the potent regulatory effects of AAL on gut inflammation suggest its substantial potential for the prevention and treatment of CRC. However, systematic research regarding its specific molecular mechanisms in CRC remains limited.

Recent studies have shown that natural anti-inflammatory agents can not only alleviate chronic inflammation, but also modulate intracellular oxidative stress and influence tumor-associated reactive oxygen species (ROS) levels (10). ROS play a crucial role in regulating cell proliferation and death in various cancers, including CRC (11). The excessive production of ROS can lead to oxidative stress, which in turn can further induce autophagy, resulting in significant cytotoxicity (12). Studies have shown that elevated levels of ROS can enhance cellular autophagy, thereby playing a role in the antitumor effect against CRC (13). Therefore, AAL may exert its antitumor effects by regulating the levels of ROS in CRC cells, thereby inducing autophagy.

In this study, we systematically analyzed the chemical composition of ethanol extracts of Acalypha australis L. (EEAL) and employed network pharmacology to identify the key bioactive components and molecular targets relevant to its therapeutic effects against CRC. Concurrently, we conducted cellular experiments to investigate the inhibitory effects of EEAL on CRC cells and explored its regulatory effects on ROS, as well as its impact on autophagy and related pathway proteins. This research aims to elucidate the antitumor efficacy of EEAL against CRC and its potential mechanisms of action. We hope that the resulting findings will provide experimental evidence and new insights for the application of traditional ethnic medicine in the prevention and treatment of CRC.

## 2 Materials and methods

### 2.1 Plant material

Fresh whole plants of AAL were purchased and identified by Deputy Chief Pharmacist Rong Tan at the Preparation Center of Guizhou University of Traditional Chinese Medicine. The plant material was washed and chopped into small segments using a grinder for extraction.

### 2.2 Sample preparation

The chopped AAL material was placed in a heated flask and immersed in ethanol. After connecting the flask, condenser, and heating apparatus, the mixture was brought to a boil, then refluxed at low heat for 2 hours. This extraction step was repeated once. The extract was filtered through a Buchner funnel and qualitative filter paper under vacuum, concentrated, and stored overnight at −20 °C, then transferred to −80 °C for overnight storage. Subsequently, the sample was freeze-dried under vacuum for 72 hours, ground into powder, and stored in a desiccator. Before use, the lyophilized powder was dissolved in complete DMEM, filtered through a 0.22 μm filter membrane, and stored at −20 °C.

### 2.3 Phytochemical analysis

The chemical constituents of the extract were accurately identified and quantified by ultra-performance liquid chromatography-tandem mass spectrometry (UPLC-MS/MS). Chromatographic conditions included an Agilent SB-C18 1.8 μm, 2.1 mm × 100 mm column, with 0.1% formic acid aqueous solution and acetonitrile as the mobile phase in gradient elution. Mass spectrometry utilized an electrospray ionization (ESI) source in both positive and negative ionization modes.

### 2.4 Network pharmacology analysis

#### 2.4.1 Screening of main active compounds

The chemical classes and contents in EEAL were determined with UHPLC-MS/MS and compared with the TCMSP database based on retention time and mass-to-charge ratio, to preliminarily identify putative active compounds.

#### 2.4.2 Swiss ADME-based screening

Canonical SMILES of the preliminarily identified compounds were obtained from PubChem and inputted into the SwissADME tool, screening for molecules with favorable drug-likeness according to Lipinski’s “Rule of Five”.

#### 2.4.3 Target prediction for active components

The SMILES or names of candidate compounds were entered into Swiss Target Prediction, which predicts possible protein targets based on structural similarity. Targets with probability > 0 were kept, duplicates removed, and targets integrated to form the candidate target set for EEAL.

#### 2.4.4 Collection of colorectal cancer-related targets

“Colorectal cancer” was used as a keyword to search GeneCards and OMIM, aggregating all related genes as a CRC disease target set.

#### 2.4.5 Construction and analysis of the compound-target-disease network

The intersection of active compound targets and CRC disease targets was derived using a Venn diagram, yielding the key “compound-disease” targets. Cytoscape software was used to construct the “compound–target–disease” network, with compounds, targets, and CRC as nodes, and their interactions as edges. Topological analysis (degree value) identified core targets.

#### 2.4.6 GO and KEGG enrichment analysis

Core target gene lists were uploaded to the Majorbio Cloud platform for GO and KEGG enrichment analysis (species: Homo sapiens, p < 0.05, q < 0.05, Top 20 displayed). Enrichment results provided functional and pathway annotation of the core targets.

### 2.5 Molecular docking

Three-dimensional structures of core target proteins were downloaded from the UniProt database; 3D structures of core active compounds from PubChem. CB-DOCK2 was used for all preprocessing steps (removal of water and endogenous ligands, hydrogenation, charge calculation, and merging nonpolar hydrogens), followed by automated docking and visualization.

### 2.6 *In vitro* experiments

#### 2.6.1 Cell culture

Human colorectal cancer cell lines HCT116 and LOVO, normal colonic epithelial cell lines FHC and NCM460 were cultured in complete DMEM at 37 °C in 5% CO_2_.

#### 2.6.2 Cell proliferation assays

The anti-proliferative effects of EEAL were assessed in HCT116, LOVO, FHC, and NCM460 cells using CCK-8 and colony formation assays. Cells (1 × 10_5_/mL) were seeded in 96-well plates, treated with different concentrations of EEAL for 24 hours, and CCK-8 reagent was used for absorbance measurement. For colony formation, pretreated HCT116, LOVO, and SW480 cells (5 × 10²/mL) were seeded in 6-well plates, media changed every three days, and colonies stained with crystal violet on day 15 after paraformaldehyde fixation.

#### 2.6.3 Mitochondrial membrane potential detection

HCT116 and LOVO cells were seeded in 6-well plates and treated with varying EEAL concentrations for 24 hours. Changes in mitochondrial membrane potential were detected using the Mito-Tracker fluorescent probe. After 20-minute incubation and Hoechst 33342 staining, red fluorescence intensity was observed by fluorescence microscopy.

#### 2.6.4 ROS level detection

Levels of intracellular ROS were detected using DCFH-DA fluorescent probe. After 24-hour EEAL treatment, cells were incubated with 10 μM DCFH-DA for 30 minutes. Fluorescence intensity was analyzed both by microscopy and flow cytometry, with quantification performed using FlowJo software.

#### 2.6.5 Cell membrane integrity (CM-Dil labeling)

HCT116 and LOVO cells (2.5 × 10_4_/mL) were seeded in confocal dishes and treated with different concentrations of EEAL. CM-Dil tracer was added (1:1000), incubated for 1 hour, washed, and observed by confocal microscopy to assess membrane integrity.

#### 2.6.6 Transmission electron microscopy

Cells are collected by centrifugation to form a pellet, followed by resuspension in fixative solution at 4 °C for 2–4 hours. After centrifugation, the supernatant is discarded, and the pellet is washed with 0.1 M phosphate buffer (pH 7.4). This step is repeated three times, with each wash lasting for 3 minutes. A 1% agarose solution is prepared by heating and is allowed to cool slightly before adding it to an EP tube. The cell pellet is then carefully picked with tweezers and suspended within the agarose before it solidifies. The pellet is fixed with 1% osmium tetroxide in 0.1 M phosphate buffer (pH 7.4) at room temperature, protected from light, for 2 hours. The buffer is washed three times for 15 minutes each. The samples are dehydrated sequentially in increasing concentrations of ethanol (30%, 50%, 70%, 80%, 95%, 100%, and 100%) for 20 minutes each, followed by two washes in 100% acetone for 15 minutes each. A mixture of acetone and embedding medium (1:1) is applied at 37 °C for 4 hours, followed by overnight infiltration with a mixture of acetone and embedding medium (1:2). Afterward, pure embedding medium is used to process samples at 37 °C for 8 hours. The liquid embedding medium is poured into embedding molds, and the samples are placed in the molds before being baked overnight at 37 °C. The embedding molds are then polymerized in an oven at 60 °C for 48 hours, after which the resin blocks are retrieved for further processing. Ultrathin sections (80 nm) are cut using an ultramicrotome, and placed on 150 mesh copper grids. The grids are stained in a dark environment with saturated alcoholic uranyl acetate for 8 minutes, followed by three washes with 70% ethanol, then three washes with ultrapure water. The sections are stained with 2.6% lead citrate solution in a CO2-free environment for 8 minutes, then washed three times with ultrapure water and gently blotted with filter paper. The copper grids are placed in a grid box and left to dry overnight at room temperature. The samples are then observed under a transmission electron microscope for imaging and analysis.

#### 2.6.7 Assessment of autophagic lysosomes using Lyso-Tracker

HCT116 and LOVO cells were cultured at a density of 1 × 10 (5) cells/ml in six-well plates for 24 hours. Subsequently, cells were treated with different concentrations of EEAL solution, adding 2 ml to each well for a 24-hour intervention. A total of 1.5 μL of Lyso-Tracker Red solution was diluted in 15 mL of high-glucose DMEM at a ratio of 1:10,000 and pre-warmed to 37 °C. After removing the cell culture medium, 1 mL of the prepared Lyso-Tracker Red staining solution was added to each well and the cells were incubated at 37 °C for 60 minutes. Following incubation, the Lyso-Tracker Red staining solution was removed, and 1 mL of fresh DMEM was added. The cells were then observed using an inverted microscope.

#### 2.6.8 Western blot analysis

HCT116 and LOVO cells (1×10_5_/mL) were treated with varying EEAL concentrations for 24 hours. Proteins were extracted with RIPA buffer, separated by SDS-PAGE, and transferred to PVDF membranes. Membranes were blocked, incubated with primary antibodies overnight at 4 °C, washed, and then incubated with HRP-conjugated secondary antibodies. Bands were visualized using enhanced chemiluminescence (ECL) and imaged on a ChemiDoc Touch system; densitometric analysis was performed with Image J.

#### 2.6.9 N-acetylcysteine rescue experiment

To elucidate the critical role of ROS in the anti-CRC effects of EEAL, a rescue experiment was performed using the ROS scavenger NAC. The experiment included four groups: blank control group, NAC alone group, NAC combined with EEAL group, and EEAL alone group. NAC was freshly prepared as a 100 mM stock solution in deionized water, sterilized by filtration through a 0.22 μm membrane, and diluted to a working concentration of 5 mM in complete DMEM immediately before use. Cells in the NAC alone group and NAC combined with EEAL group were pretreated with 5 mM NAC for 2 h. Subsequently, cells in the NAC combined with EEAL group were incubated with fresh medium containing the indicated concentrations of EEAL for an additional 24 h, while cells in the NAC alone group were incubated with fresh medium for 24 h. Cells in the blank control group and EEAL alone group were not pretreated with NAC; cells in the EEAL alone group were directly treated with EEAL for 24 h, and cells in the blank control group were incubated with fresh medium for 24 h.

Intracellular ROS levels were detected using the DCFH-DA fluorescent probe. After 24 h of treatment according to the groups described above, HCT116 and LOVO cells were incubated with 10μM DCFH-DA at 37 °C for 30 min in the dark. Fluorescence intensity was analyzed by flow cytometry, and quantification was performed using FlowJo software.

Cell viability was assessed using the CCK-8 assay. HCT116 and LOVO cells were seeded in 96-well plates and treated according to the groups described above for 24 h. Subsequently, 10μL of CCK-8 solution was added to each well and incubated at 37 °C for 2 h. Absorbance was measured at 450 nm using a microplate reader, and cell viability was expressed as a percentage relative to the blank control group.

LC3B protein expression levels were detected by Western blot. After 24 h of treatment according to the groups described above, HCT116 and LOVO cells were harvested and total protein was extracted using RIPA lysis buffer. Protein samples were separated by SDS-PAGE, transferred to PVDF membranes, and incubated with primary antibodies against LC3B and GAPDH overnight at 4 °C, followed by incubation with HRP-conjugated secondary antibodies at room temperature for 1 h. Protein bands were visualized using ECL reagent and imaged on a ChemiDoc Touch system. Densitometric analysis was performed using ImageJ software, and LC3B expression levels were normalized to GAPDH.

### 2.7 Statistical analysis

All data are presented as the mean ± standard error of the mean from three independent experiments. Statistical comparisons between multiple groups were performed using one-way analysis of variance (ANOVA) followed by Dunnett’s *post-hoc* test. Data visualization was performed using GraphPad Prism version 6.0. A p-value of less than 0.05 was considered statistically significant.

## 3 Results

### 3.1 Screening of active compounds and targets

**Figure 1:**
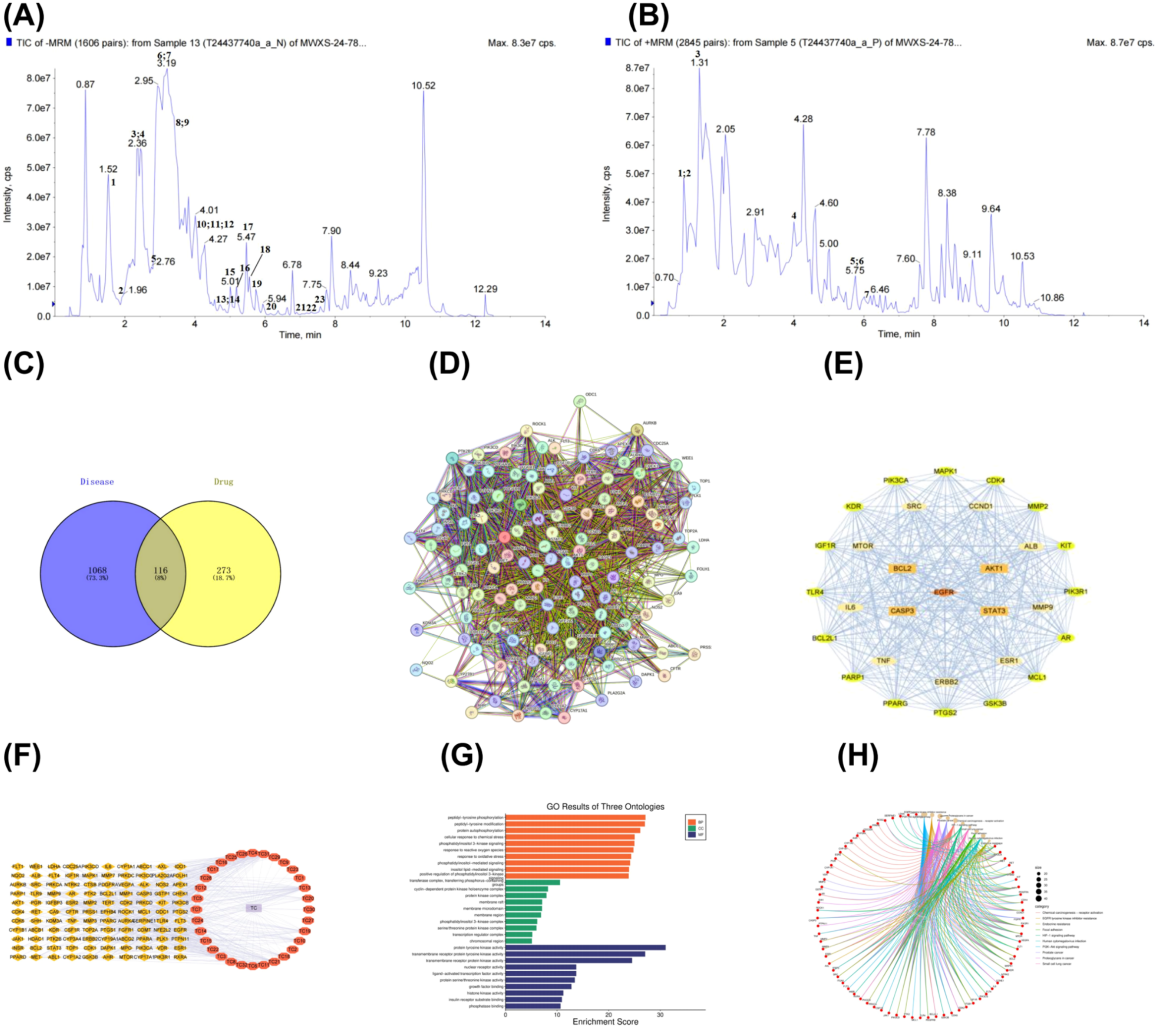
Bioactive compounds of EEAL and potential therapeutic targets for colorectal cancer (CRC), with GO and KEGG enrichment analyses. **(A)** Total ion chromatogram of EEAL in positive ion mode by UHPLC-MS/MS. **(B)** Total ion chromatogram of EEAL in negative ion mode by UHPLC-MS/MS. **(C)** Venn diagram of overlapping targets. **(D)** PPI (protein–protein interaction) network. **(E)** Core therapeutic targets for CRC identified by Cytoscape analysis. **(F)** “Drug–compound–target” interaction network: orange indicates targets, orange-red represents compounds, and light purple denotes the drug EEAL. **(G)** GO enrichment analysis of EEAL against CRC, including CC (cellular component), BP (biological process), and MF (molecular function) categories. **(H)** KEGG pathway analysis.

Based on UHPLC-MS analysis (**Figures 1A, B**), a total of 30 bioactive components of EEAL with potential pharmacological significance were identified through comprehensive screening using the TCMSP, Herb, SwissTargetPrediction, and PubChem databases. Among these, phenolic acids accounted for 43%, flavonoids for 20%, organic acids for 10%, terpenoids for 7%, alkaloids for 7%, quinones for 3%, and other compounds for 10%. The identified phenolic acids included: ethyl gallate, 3,5-dihydroxy-4-methoxybenzoic acid, gallic acid, caffeic acid, methyl p-hydroxybenzoate, octyl gallate, methyl gallate, 3-O-methylgallic acid, methyl protocatechuate, ethyl 3,4-dihydroxybenzoate, protocatechuic acid, 3-methylsalicylic acid, and caffeic acid ethyl ester. The identified flavonoids were: 3,7-di-O-methylquercetin, 8-methoxyapigenin, quercetin 3,4’-dimethyl ether, apigenin, quercetin, and naringenin. The identified quinone was: ω-hydroxyemodin. The identified alkaloids were: methyl nicotinate and 5-methylnicotinic acid. The identified terpenoids included: 2,3,19,23-tetrahydroxyurs-12-en-28-oic acid and 2,3,23-trihydroxyolean-12-en-28-oic acid. Organic acids identified were: 2-propylsuccinic acid, 2-benzylsuccinic acid, and monomethyl succinate. Other identified compounds included: caffeic acid 3’-sulfate, isonicotinic acid, and niacin (vitamin B3) (**Table 1**). These 30 compounds were mapped to a total of 273 potential targets. Meanwhile, 1,068 CRC-related targets were retrieved from the GeneCards and OMIM databases. The intersection between the compound-related and disease-related targets yielded 116 common targets (**Figure 1C**). The PPI network, generated by the STRING database (**Figure 1D**) and further analyzed using Cytoscape software (**Figure 1F**), revealed a “drug–compound–target” interactive network. Core targets such as EGFR, BCL2, and CASP3 (**Figure 1E**) were highlighted, suggesting that EEAL may induce apoptosis in colorectal cancer cells through the regulation of mitochondrial membrane potential permeability.

**Table 1:** UHPLC-MS/MS analysis results.

Compounds	Class I	Class II	Molecular weight (Da)	Formula	Ionization model
Gallic Acid Ethyl Ester	Phenolic acids	Phenolic acids	198.0528	C_9_H_10_O_5_	[M-H]-
3,5-Dihydroxy-4-methoxybenzoic acid	Phenolic acids	Phenolic acids	184.0372	C_8_H_8_O_5_	[M-H]-
Gallate	Phenolic acids	Phenolic acids	170.0215	C_7_H_6_O_5_	[M-H]-
Caffeate	Phenolic acids	Phenolic acids	180.0423	C_9_H_8_O_4_	[M-H]-
Methyl 4-hydroxybenzoate	Phenolic acids	Phenolic acids	152.0473	C_8_H_8_O_3_	[M-H]-
Octyl gallate	Phenolic acids	Phenolic acids	282.1467	C_15_H_22_O_5_	[M-H]-
Methyl gallate	Phenolic acids	Phenolic acids	184.0372	C_8_H_8_O_5_	[M-H]-
3-O-Methylgallate	Phenolic acids	Phenolic acids	184.0372	C_8_H_8_O_5_	[M-H]-
Protocatechuic Acid Methyl Ester	Phenolic acids	Phenolic acids	168.0423	C_8_H_8_O_4_	[M-H]-
3,4-Dihydroxybenzoic Acid Ethyl Ester	Phenolic acids	Phenolic acids	182.0579	C_9_H_10_O_4_	[M-H]-
3,4-Dihydroxybenzoate	Phenolic acids	Phenolic acids	154.0266	C_7_H_6_O_4_	[M-H]-
3-Methylsalicylate	Phenolic acids	Phenolic acids	152.0473	C_8_H_8_O_3_	[M-H]-
Ethyl caffeate	Phenolic acids	Phenolic acids	208.0736	C_11_H_12_O_4_	[M-H]-
3,7-Di-O-methylquercetin	Flavonoids	Flavonols	330.074	C_17_H_14_O_7_	[M-H]-
8-Methoxyapigenin	Flavonoids	Flavones	300.0634	C_16_H_12_O_6_	[M+H]+
Quercetin 3,4’-dimethyl ether	Flavonoids	Flavonols	330.074	C_17_H_14_O_7_	[M+H]+
Apigenin	Flavonoids	Flavones	270.0528	C_15_H_10_O_5_	[M+H]+
Quercetin	Flavonoids	Flavonols	302.0427	C_15_H_10_O_7_	[M-H]-
Naringenin	Flavonoids	Flavanones	272.0685	C_15_H_12_O_5_	[M-H]-
Citreorosein	Quinones	Anthraquinone	286.0477	C_15_H_10_O_6_	[M-H]-
Caffeic acid 3’-sulfate	Others	Others	259.9991	C_9_H_8_O_7_S	[M-H]-
Isonicotinic acid	Others	Vitamin	123.032	C_6_H_5_NO_2_	[M+H]+
Nicotinate	Others	Vitamin	123.032	C_6_H_5_NO_2_	[M+H]+
Nicotinic Acid Methyl Ester	Alkaloids	Pyridine alkaloids	137.0477	C_7_H_7_NO_2_	[M+H]+
5-Methylnicotinic acid	Alkaloids	Alkaloids	137.0477	C_7_H_7_NO_2_	[M+H]+
2,3,19,23-Tetrahydroxyurs-12-en-28-oic acid	Terpenoids	Triterpene	504.3451	C_30_H_48_O_6_	[M-H]-
2,3,23-Trihydroxyolean-12-en-28-oic acid	Terpenoids	Triterpene	488.3502	C_30_H_48_O_5_	[M-H]-
2-Propylsuccinic acid	Organic acids	Organic acids	160.0736	C_7_H_12_O_4_	[M-H]-
2-Benzylsuccinic Acid	Organic acids	Organic acids	208.0736	C_11_H_12_O_4_	[M-H]-

### 3.2 Pathway enrichment analysis of EEAL in the treatment of CRC

GO analysis (**Figure 1G**) revealed that EEAL are associated with the induction of oxidative stress responses. Previous studies have shown that alterations in oxidative stress tolerance can lead to mitochondrial changes and subsequently promote the induction of apoptosis. KEGG pathway analysis (**Figure 1H**) indicated that the underlying mechanisms may involve the PI3K-Akt, HIF-1, and other related signaling pathways.

### 3.3 Molecular docking validation

**Figure 2:**
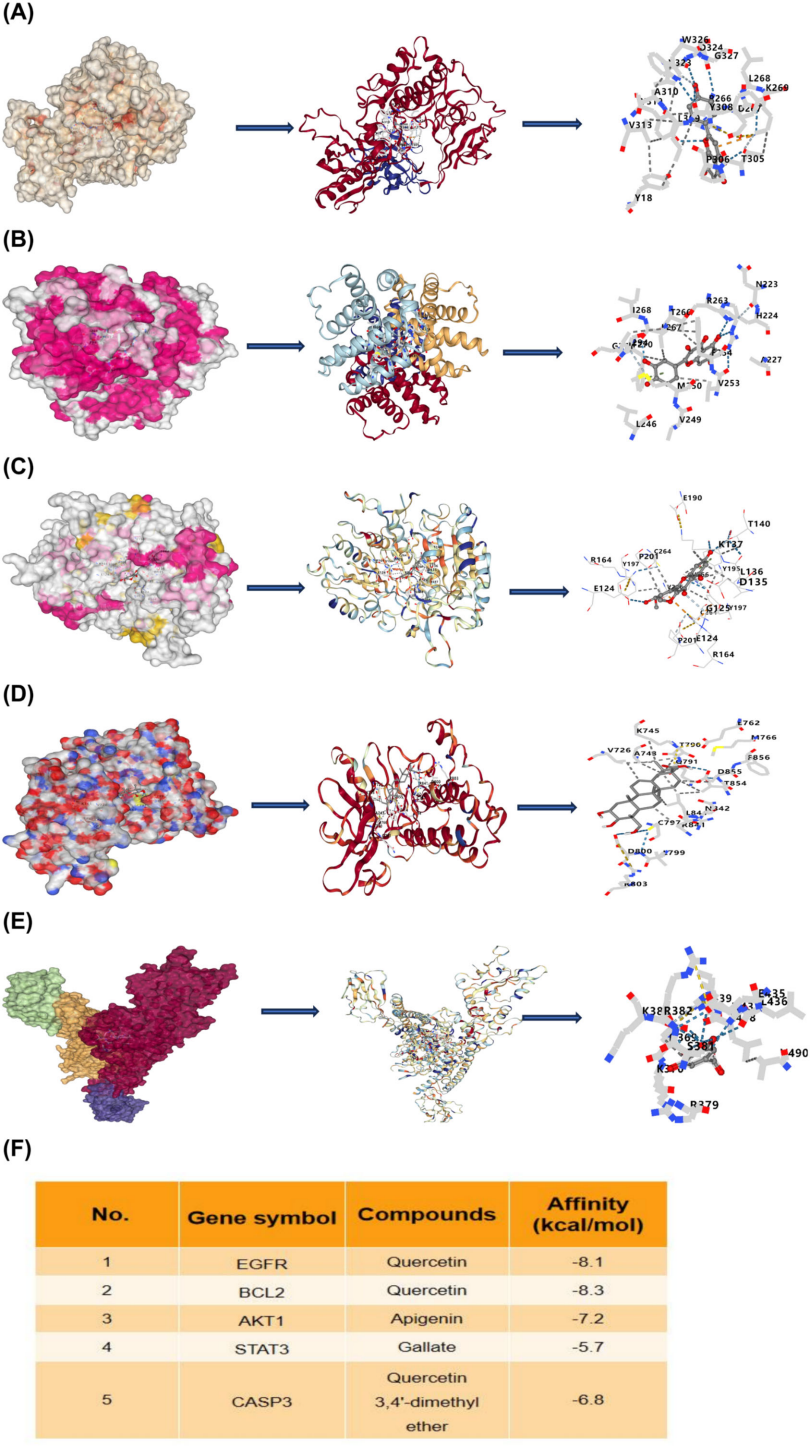
Molecular docking visualization analysis. **(A)** Docking of EGFR with quercetin. **(B)** Docking of BCL-2 with quercetin. **(C)** Docking of AKT1 with apigenin. **(D)** Docking of STAT3 with gallate. **(E)** Docking of CASP3 with quercetin 3,4’-dimethyl ether. **(F)** Binding energies of core compounds with target proteins.

Based on the PPI network, the top five target proteins of EEAL were identified by degree value (**Figures 2A–E**). The binding energies between the core compounds and their target proteins were all less than −5.0 kcal/mol (**Figure 2F**), indicating stable interactions.

### 3.4 *In vitro* experiments

#### 3.4.1 Effects of EEAL on the proliferative activity of colorectal cancer cells

**Figure 3:**
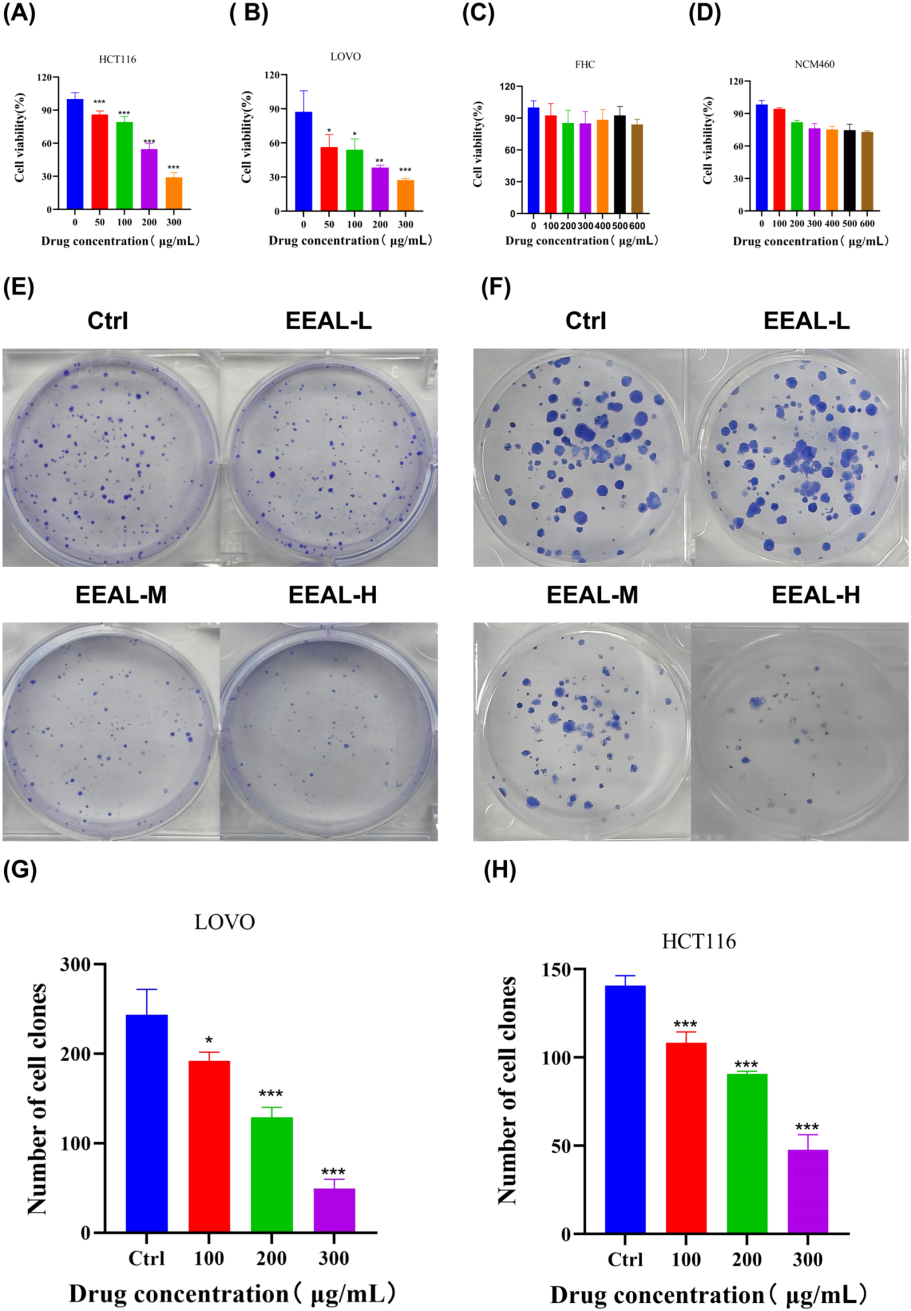
EEAL inhibits the proliferative capacity of colorectal cancer cells. **(A)** CCK8 assay results of HCT116 cells after 24 hours of EEAL treatment. **(B)** CCK8 assay results of LOVO cells after 24 hours of EEAL treatment. **(C)** CCK8 assay results of FHC cells after 24 hours of EEAL treatment. **(D)** CCK8 assay results of NCM460 cells after 24 hours of EEAL treatment. **(E)** Colony formation results of HCT116 cells. **(F)** Colony formation results of LOVO cells. **(G)** Quantitative analysis of colony formation in LOVO cells. **(H)** Quantitative analysis of colony formation in HCT116 cells. All data are presented as mean ± SD from three independent experiments. Statistical significance was determined by one-way ANOVA followed by Dunnettde *post hoc* test. (*P < 0.05, **P < 0.01, ***P < 0.001, ****P < 0.0001 vs. control group).

CCK8 assay results demonstrated that EEAL at concentrations of 50–300 μg/ml significantly inhibited the proliferation of colorectal cancer cell lines HCT116 and LOVO (P < 0.001), with both cell lines exhibiting a clear dose-dependent response (**Figures 3A, B**). Prism analysis revealed that the IC_50_ for HCT116 cells was 170.9 μg/ml, whereas for LOVO cells it was 114.5 μg/ml. In contrast, EEAL treatment at concentrations up to 600 μg/ml showed no significant inhibition of proliferation in FHC cells (**Figure 3C**), and no apparent effect was observed on NCM460 cell proliferation (**Figure 3D**).

Further, colony formation assays in HCT116 and LOVO cells indicated a marked, dose-dependent inhibition of proliferative capacity following EEAL treatment (**Figures 3E, F**). In control groups, numerous dense colonies with full morphology were observed. ImageJ analysis demonstrated that EEAL treatment significantly decreased colony numbers in a statistically significant manner, *P* < 0.001. (**Figures 3G, H**), confirming the substantial and concentration-dependent inhibitory effect of EEAL on colorectal cancer cell proliferation and colony formation.

#### 3.4.2 EEAL disrupts tumor cell membrane integrity, reduces mitochondrial membrane potential, and increases intracellular ROS levels

**Figure 4:**
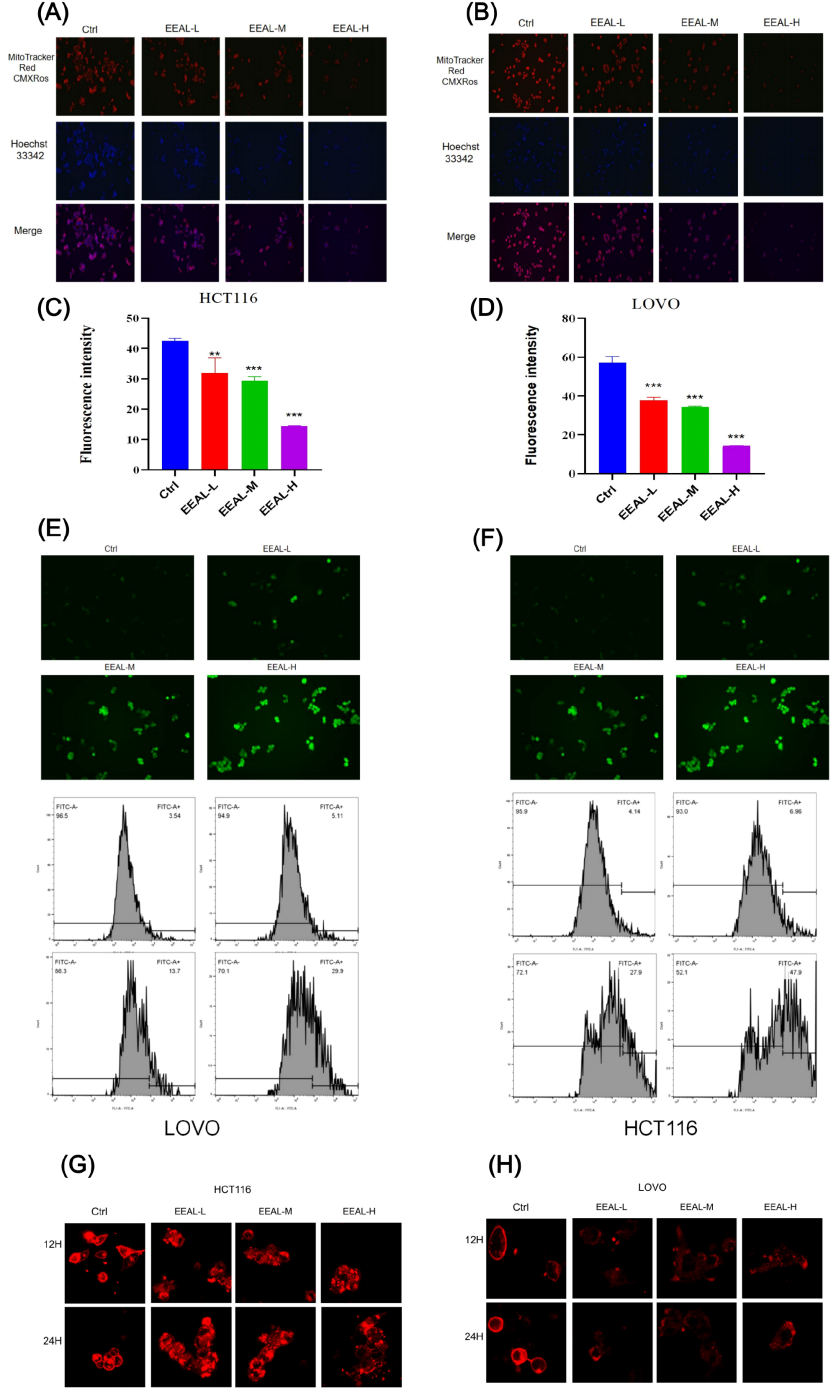
**(A)** Mitochondrial membrane potential fluorescence images of HCT116 cells. **(B)** Mitochondrial membrane potential fluorescence images of LOVO cells. **(C)** Quantitative analysis of mitochondrial membrane potential in HCT116 cells. **(D)** Quantitative analysis of mitochondrial membrane potential in LOVO cells. **(E)** EEAL treatment for 24 hours increases intracellular ROS levels in LOVO cells. **(F)** EEAL treatment for 24 hours increases intracellular ROS levels in HCT116 cells. **(G)** After 12 and 24 hours of EEAL treatment, different degrees of membrane rupture are observed in HCT116 cells. **(H)** After 12 and 24 hours of EEAL treatment, different degrees of membrane rupture are observed in LOVO cells. All data are presented as mean ± SD from three independent experiments. Statistical significance was determined by one-way ANOVA followed by Dunnettde *post hoc* test. (*P < 0.05, **P < 0.01, ***P < 0.001, ****P < 0.0001 vs. control group).

Mito-Tracker staining showed that control groups of both colorectal cancer cell lines displayed high mitochondrial membrane potential, as evidenced by strong red fluorescence signals, indicative of intact mitochondrial function. However, treatment with increasing concentrations of EEAL resulted in a marked decline in mitochondrial membrane potential, displayed as progressively diminished red fluorescence intensity (**Figures 4A, B**). This reduction was quantitatively confirmed using ImageJ (**Figures 4C, D**).

In the control groups, baseline ROS levels were low, and only faint fluorescence was observed under the microscope. Post-EEAL treatment, intracellular ROS levels rose significantly, with fluorescence intensity increasing in a dose-dependent manner (**Figures 4E, F**). Flow cytometric analysis further quantified the increase in ROS-positive cells: in HCT116 controls, the proportion was 4.14%, increasing to 6.96%, 27.9%, and 47.9% with EEAL concentrations of 100, 150, and 200 μg/ml, respectively. LOVO cells showed a similar trend, with ROS-positive cell proportions of 3.54% (control), 5.11% (50 μg/ml), 13.7% (150 μg/ml), and 29.9% (250 μg/ml).

These data directly demonstrate that EEAL robustly induces ROS production in both cell lines in a concentration-dependent manner. Additionally, membrane permeability analysis using the CM-Dil kit (**Figures 4G, H**) revealed that EEAL treatment compromised tumor cell membrane integrity, as evidenced by the diffusion of red fluorescence, confirming loss of membrane stability and release of intracellular components.

#### 3.4.3 EEAL induces overactivation of intracellular autophagic lysosomes

**Figure 5:**
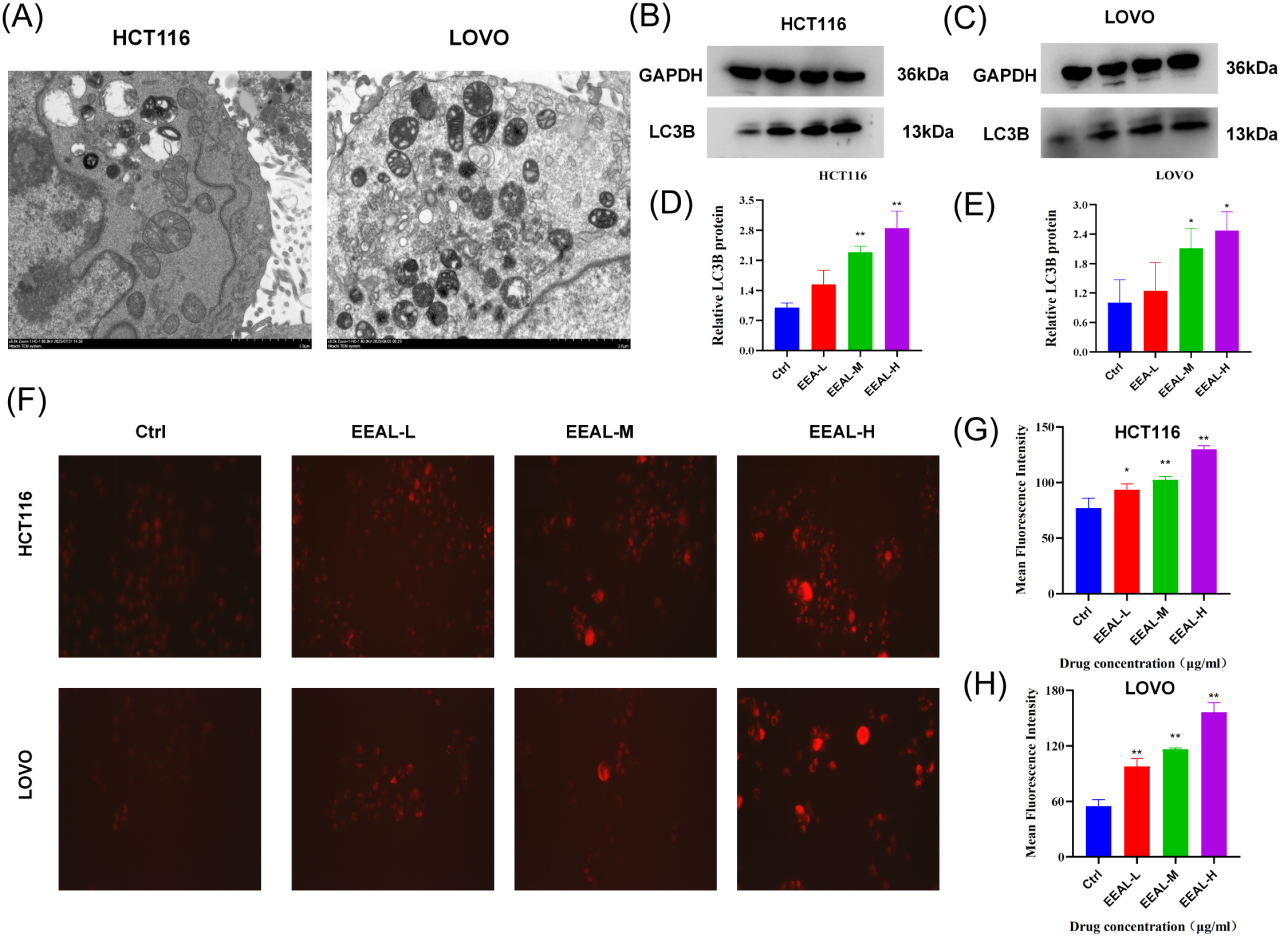
**(A)** Transmission electron microscopy images of HCT116 and LOVO cells; **(B–E)** Representative images of LC3B in HCT116 and LOVO cells along with corresponding protein expression levels; **(F)** Fluorescence images of Lyso-Tracker staining in HCT116 and LOVO cells; **(G)** Analysis of Lyso-Tracker staining fluorescence intensity in HCT116 cells; **(H)** Analysis of Lyso-Tracker staining fluorescence intensity in LOVO cells. All data are presented as mean ± SD from three independent experiments. Statistical significance was determined by one-way ANOVA followed by Dunnettde *post hoc* test. (*P < 0.05, **P < 0.01, ***P < 0.001, ****P < 0.0001 vs. control group).

HCT116 and LOVO cells were treated with culture media containing EEAL at concentrations of 200 µg/ml and 150 µg/ml, respectively, and subsequently assessed using transmission electron microscopy. Following EEAL treatment, a significant accumulation of autophagic lysosomes was observed in both cell lines (**Figure 5A**). Additionally, Lyso-Tracker staining revealed that the red fluorescence intensity gradually increased with higher drug concentrations (**Figure 5F**), indicating an increase in intracellular autophagic lysosomes. Statistical analysis of fluorescence intensity showed significant differences (P < 0.05), confirming that EEAL leads to an increase in autophagic lysosomes in both cell types, exhibiting a clear dose-dependent effect (**Figures 5G, H**). Western blot analysis of the autophagy marker LC3B demonstrated a trend of upregulation with statistical significance (**Figures 5B–E**).

#### 3.4.4 EEAL inhibits the expression of AKT/mTOR/HIF-1 proteins

**Figure 6:**
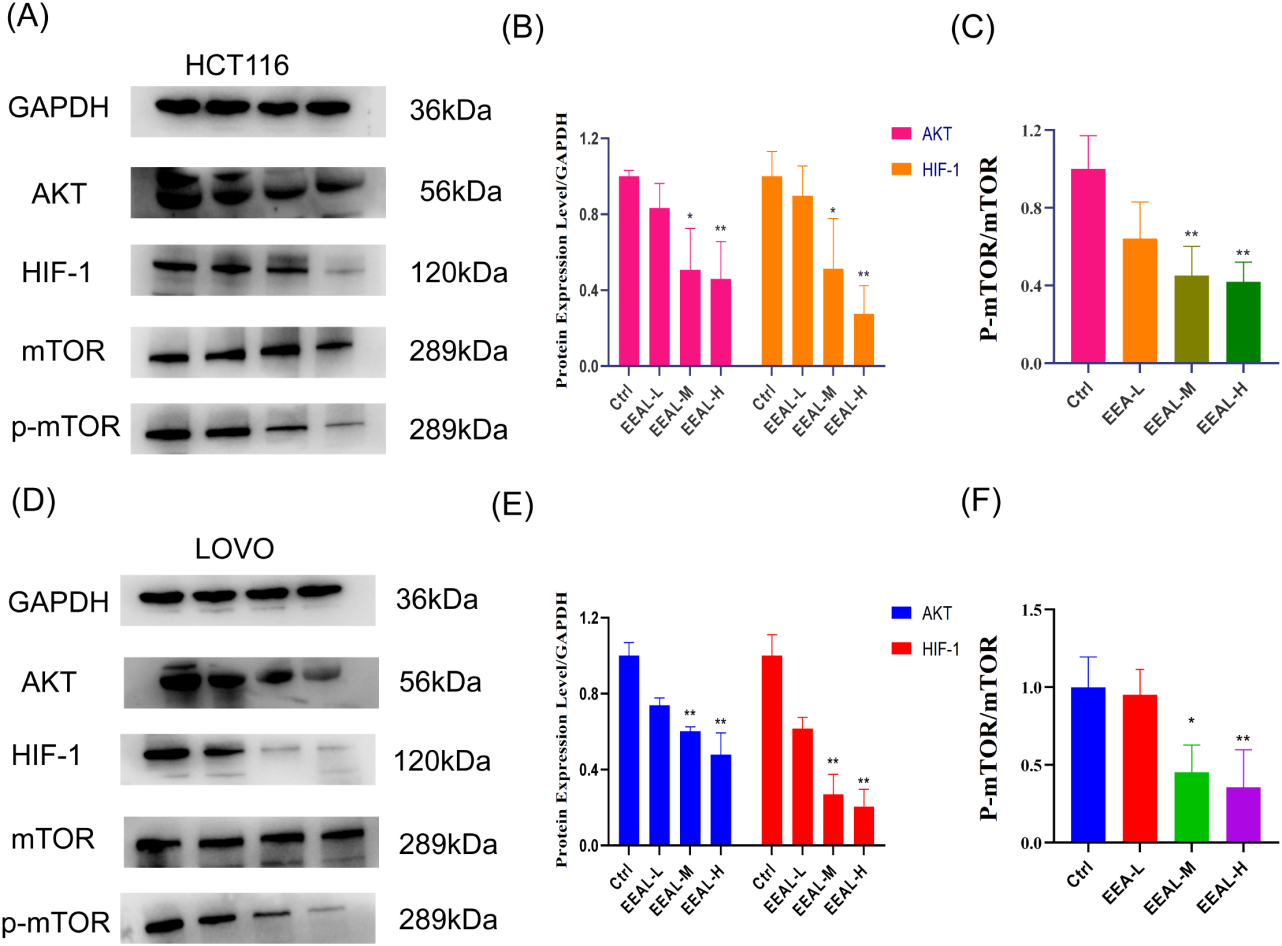
**(A)** Representative images of the inhibition of AKT/mTOR/HIF-1 protein expression in HCT116 cells; **(B)** Quantitative analysis of AKT and HIF-1 protein levels in HCT116 cells; **(C)** Quantitative analysis of P-mTOR protein levels in HCT116 cells; **(D)** Representative images of the inhibition of AKT/mTOR/HIF-1 protein expression in LOVO cells; **(E)** Quantitative analysis of AKT and HIF-1 protein levels in LOVO cells; **(F)** Quantitative analysis of P-mTOR protein levels in LOVO cells. All data are presented as mean ± SD from three independent experiments. Statistical significance was determined by one-way ANOVA followed by Dunnettde *post hoc* test. (*P < 0.05, **P < 0.01, ***P < 0.001, ****P < 0.0001 vs. control group).

We investigated the regulatory effect of EEAL on the AKT/mTOR/HIF-1 signaling pathway in HCT116 and LOVO cells. As shown in **Figure 6**, treatment with various concentrations of EEAL resulted in a concentration-dependent decrease in the protein expression levels of AKT, HIF-1, and phosphorylated mTOR (p-mTOR) in both HCT116 (**Figure 6A**) and LOVO (**Figure 6D**) cells, while the expression of the housekeeping protein GAPDH remained unchanged. Further quantitative analysis (**Figures 6B, E**) indicated that, compared to the control group, the expression levels of AKT and HIF-1 in HCT116 cells were significantly reduced following treatment with medium and high concentrations of EEAL (P < 0.05). In LOVO cells, a similar trend in the downregulation of these proteins was observed. Additionally, to clarify the changes in mTOR activity, we analyzed the ratio of p-mTOR to total mTOR (**Figures 6C, F**). In both cell lines, EEAL treatment significantly reduced this ratio, suggesting that EEAL inhibits the phosphorylation and activation of mTOR. Collectively, these findings indicate that EEAL downregulates the expression of AKT, thereby suppressing mTOR activation and downstream HIF-1 expression, ultimately blocking the transmission of the AKT/mTOR/HIF-1 signaling pathway.

#### 3.4.5 NAC attenuates EEAL-induced ROS elevation, autophagy activation, and cell viability reduction in colorectal cancer cells

To validate the critical role of ROS in EEAL-induced autophagic cell death in colorectal cancer cells, a rescue experiment was performed using the ROS scavenger NAC. The experiment included four groups: blank control, NAC alone, NAC combined with EEAL, and EEAL alone.

**Figure 7:**
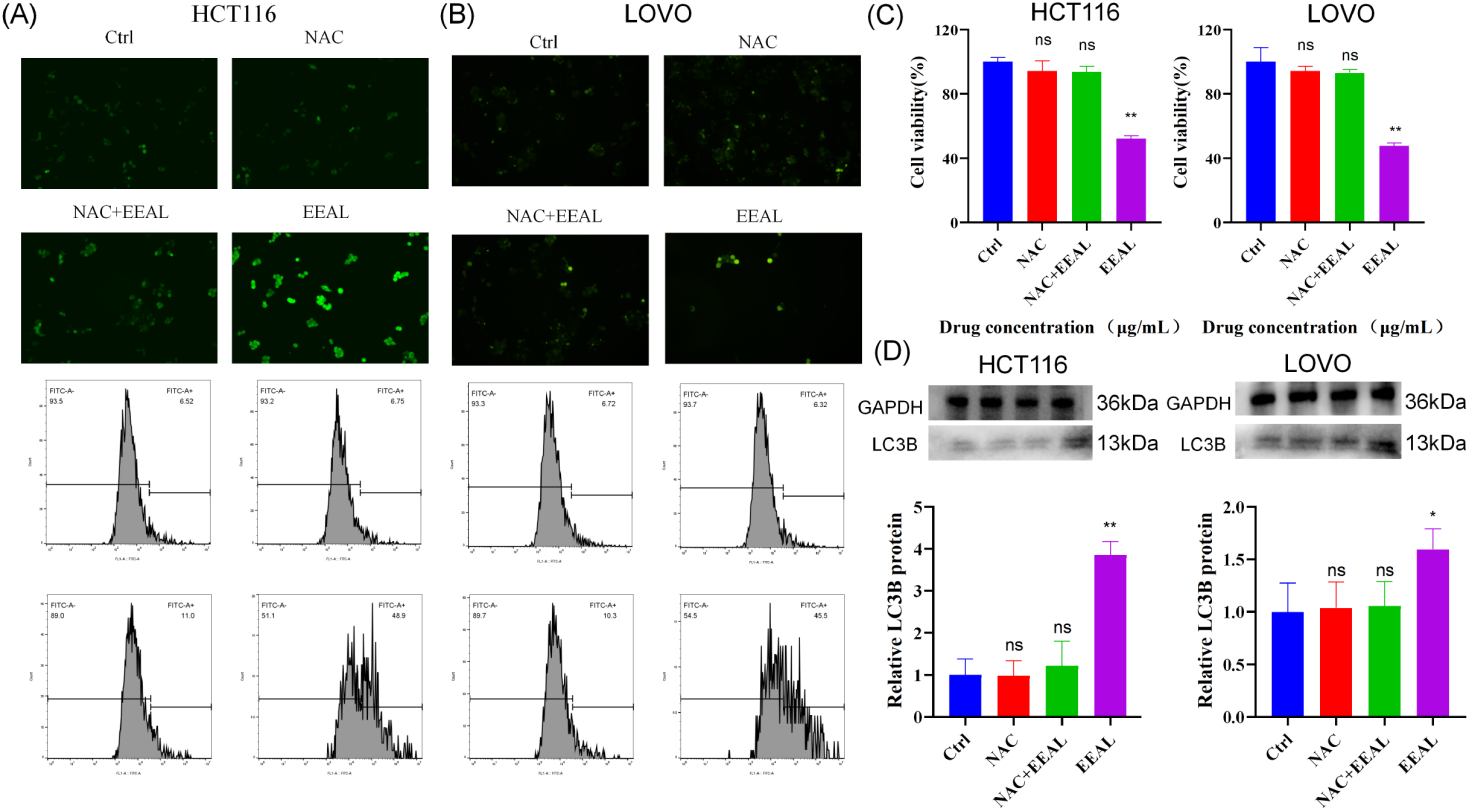
**(A)** NAC combined with EEAL treatment for 24 hours increases intracellular ROS levels in HCT116 cells. **(B)** NAC combined with EEAL treatment for 24 hours increases intracellular ROS levels in LOVO cells. **(C)** CCK8 assay results of HCT116 and LOVO cells after 24 hours of NAC combined with EEAL treatment. **(D)** Representative images and protein levels of LC3B in HCT116 and LOVO cells after NAC combined with EEAL treatment. All data are presented as mean ± SD from three independent experiments. Statistical significance was determined by one-way ANOVA followed by Dunnettde *post hoc* test. (*P < 0.05, **P < 0.01, ***P < 0.001, ****P < 0.0001 vs. control group).

Observation under an inverted microscope revealed that fluorescence intensity in the NAC combined with EEAL group was markedly diminished compared to the EEAL alone group. Flow cytometric analysis showed that baseline intracellular ROS levels were low in the control group, with ROS-positive cell proportions of 6.52% in HCT116 cells and 6.72% in LOVO cells. EEAL treatment alone significantly elevated intracellular ROS levels, with the proportion of ROS-positive cells increasing to 48.9% in HCT116 cells and 45.5% in LOVO cells following EEAL treatment. Notably, NAC pretreatment markedly attenuated EEAL-induced ROS elevation, as the ROS-positive cell proportion in the NAC combined with EEAL group was substantially reduced compared to the EEAL alone group, approaching control levels. No significant difference in ROS levels was observed between the NAC alone group and the blank control group (**Figures 7A, B**).

CCK-8 cell viability assays demonstrated that EEAL treatment alone significantly reduced the viability of colorectal cancer cells compared to the control group. However, NAC pretreatment markedly attenuated the cytotoxic effects of EEAL, as cell viability in the NAC combined with EEAL group was significantly restored compared to the EEAL alone group. No significant difference in cell viability was observed between the NAC alone group and the blank control group (**Figure 7C**).

Western blot analysis revealed that LC3B protein expression was maintained at basal levels in the control group. EEAL treatment alone significantly upregulated the expression of the autophagy marker protein LC3B compared to the control group. Importantly, NAC pretreatment effectively reversed EEAL-induced LC3B expression changes, with LC3B levels in the NAC combined with EEAL group significantly decreased compared to the EEAL alone group. LC3B expression in the NAC alone group was comparable to that of the blank control group (**Figure 7D**).

These results confirm that ROS scavenging effectively blocks EEAL-induced autophagic flux and rescues the resulting reduction in cell viability, demonstrating that ROS serves as a critical signaling molecule mediating EEAL-induced autophagic cell death in colorectal cancer cells.

## 4 Discussion

**Figure 8:**
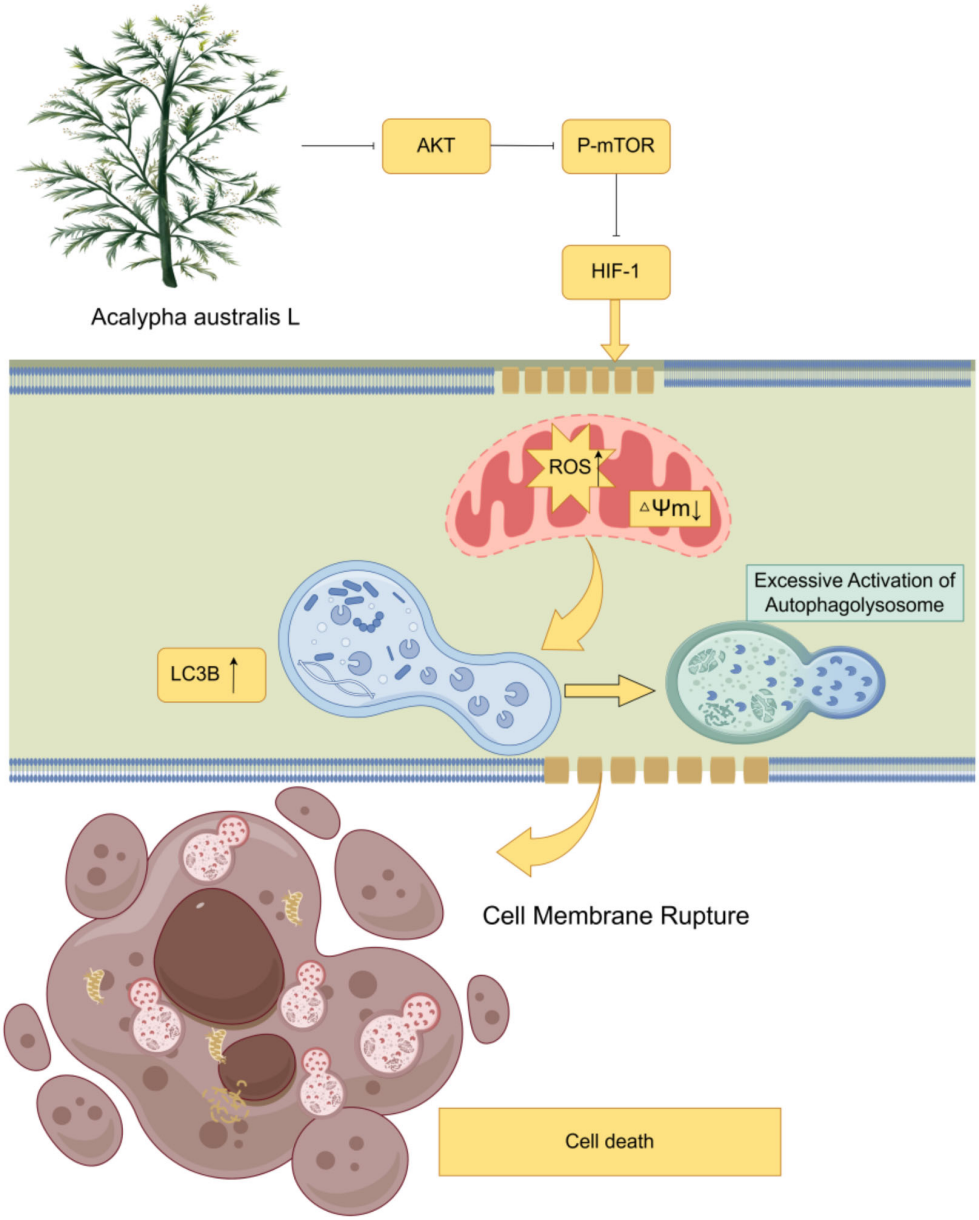
Schematic illustration of the mechanism by which EEAL exerts its anti-colorectal cancer (CRC) effects.

Our research reveals that treatment with EEAL significantly inhibits the activation of the AKT/mTOR/HIF-1 signaling pathway in CRC cells, with the expression levels of key proteins decreasing in a concentration-dependent manner. Concurrently, intracellular levels of ROS markedly increased, while mitochondrial membrane potential exhibited a concentration-dependent decline, indicating mitochondrial dysfunction. The accumulation of ROS and the resultant mitochondrial abnormalities may have induced the overactivation of the autophagic lysosome system, characterized by an increased number of autophagic lysosomes, elevated expression of LC3B protein, and enhanced autophagic flux. Ultimately, this excessive autophagic process surpasses the adaptive capacity of the cells, leading to membrane rupture and subsequent cell death in CRC cells. Therefore, EEAL may exert its anti-CRC effects by regulating the AKT/mTOR/HIF-1 pathway and the autophagic lysosome system (**Figure 8**).

First, chemical composition analysis and database screening revealed that EEAL is enriched with bioactive constituents, such as quercetin and kaempferol, both known for their anti-inflammatory and antitumor activities. Previous studies have demonstrated that these natural compounds not only scavenge free radicals (14, 15)and suppress inflammatory responses (16, 17)but also regulate intracellular signaling pathways relevant to CRC initiation and progression (18, 19). Network pharmacology further predicts that EEAL may exert its anti- CRC effects by modulating cellular oxidative stress. Previous studies have demonstrated that bioactive compounds such as quercetin (20, 21) and kaempferol (15, 22) can exert anticancer effects by regulating ROS, providing a theoretical basis for our research.

In functional assays, EEAL significantly inhibited the viability and colony formation ability of LOVO and HCT116 CRC cell lines. These inhibitory effects are consistent with both the predicted activities of its chemical constituents and current trends in natural product-based antitumor research. Numerous prior reports have confirmed that natural compounds exert unique advantages in solid tumor therapy, including CRC, by acting on multiple targets and pathways simultaneously (23, 24).

Mechanistically, our data indicate that EEAL treatment leads to a marked reduction in mitochondrial membrane potential and a significant increase in intracellular ROS levels in CRC cells, suggestive of mitochondrial dysfunction and enhanced oxidative stress. Although the main active components of AAL, such as quercetin and kaempferol, demonstrate significant antioxidant activity by eliminating ROS, some studies have also found that these antioxidants can increase intracellular ROS levels to exert their anticancer effects (21, 22, 25). Our study also revealed that EEAL significantly increases the accumulation of autolysosomes in CRC cells, suggesting that EEAL greatly enhances the autophagic capacity of CRC cells. Additionally, we observed that EEAL disrupts the membrane integrity of CRC cells. Autophagy is a self-protective mechanism in cells, primarily responsible for the degradation and recycling of unnecessary or damaged cellular components. This process is crucial for maintaining cellular homeostasis, responding to nutrient deprivation, eliminating harmful substances, and, to some extent, combating diseases such as cancer (26). However, numerous studies have demonstrated that excessive autophagy can also lead to cell death, including in cancer cells (27, 28). The increase in ROS typically activates autophagy as a cellular response to oxidative stress (29). Therefore, our findings suggest that EEAL may induce autophagic cell death in CRC cells by increasing intracellular ROS levels.

Our network pharmacology study suggests that EEAL may induce autophagic cell death in CRC cells through the AKT/mTOR/HIF-1 signaling pathway. Further validation via WB analysis indicated that EEAL can suppress the expression of AKT and downregulate the phosphorylation of mTOR, while also reducing the expression of HIF-1. Additionally, the autophagy marker LC3B exhibited an upward trend. The results of the WB experiments further confirm that EEAL induces autophagic cell death in CRC cells via the AKT/mTOR/HIF-1 pathway. Numerous previous studies have found that inhibition of the AKT/mTOR pathway induces autophagic cell death in cancer cells (30, 31). Furthermore, previous studies have shown that inhibiting the AKT/mTOR pathway can downregulate the expression of HIF-1 (32). The suppression of HIF-1 expression is associated with an increase in intracellular ROS levels (33). Elevated ROS can promote cellular autophagy, ultimately leading to an increase in the expression of the autophagy marker LC3B. These prior studies support the reliability of our research findings.

To verify the results of the aforementioned related experiments and prove that EEAL can upregulate ROS-induced autophagic cell death in CRC, we also conducted an NAC rescue experiment. In the present study, the NAC rescue experiment further established the central regulatory role of ROS in EEAL-induced autophagic cell death in CRC cells. Our results demonstrated that NAC pretreatment significantly inhibited EEAL-induced ROS elevation, while effectively reversing both the upregulation of LC3B expression and the reduction in cell viability. This finding carries important mechanistic implications. First, it confirms the identity of ROS as an upstream signaling molecule. Although our preliminary experiments observed that EEAL simultaneously induced ROS accumulation and autophagy activation, the causal relationship between them remained unclear—whether ROS is a concomitant phenomenon or a triggering factor of autophagy has been a focal point in the field of cell death research. By introducing the specific ROS scavenger NAC, we found that ROS scavenging not only blocked autophagic flux activation but also rescued cell viability, providing direct evidence that ROS acts upstream of autophagy. our results further support the dynamic balance theory of “moderate autophagy promotes survival, excessive autophagy promotes death.” In the EEAL-alone group, excessive autophagy activation was accompanied by a significant decrease in cell viability; following NAC intervention, autophagy levels returned to near-basal states, and cell survival correspondingly recovered. This phenomenon suggests that EEAL-induced ROS accumulation disrupts cellular homeostasis, shifting autophagy from an adaptive survival mechanism to a cell death execution pathway. This ROS-driven autophagic death may represent the ultimate fate choice of tumor cells under conditions of oxidative stress overload. Furthermore, our findings provide a methodological reference for mechanistic studies of natural product antitumor effects. In natural product research, due to the complexity of extract components, the respective contributions of single compounds versus mixtures are often difficult to distinguish. Through the rescue experiment strategy, this study functionally validated the necessity of the key node ROS, laying a solid foundation for subsequent bioactive component tracing and in-depth exploration of mechanisms. Future studies may further combine RNA interference technology to knock down or overexpress autophagy key genes (such as ATG5, Beclin1) to more comprehensively elucidate the molecular regulatory network of EEAL-induced autophagic death. In summary, the NAC rescue experiment not only clarified the causal role of ROS in the mechanism of EEAL action but also revealed the functional link between oxidative stress and autophagic death, providing a theoretical basis for developing EEAL as a candidate anti-CRC drug targeting the ROS/autophagy axis.

Our findings provide the first evidence that Acalypha australis L. induces autophagic cell death in colorectal cancer cells by increasing ROS through the suppression of the AKT/mTOR/HIF-1 pathway. However, our study has several limitations that must be acknowledged. First, this research has only demonstrated the anticancer effects of EEAL at the cellular level; further studies are necessary to isolate, purify, and compare individual active components. Second, there is a lack of *in vivo* efficacy and safety data, which must be addressed in future animal and clinical studies to clarify pharmacokinetics, toxicity, and long-term safety. Third, network pharmacology analysis highlighted several core targets for EEAL against CRC, including EGFR, BCL2, and CASP3, alongside the AKT/mTOR axis. While our experimental validation focused on the AKT/mTOR/HIF-1 pathway due to its central role in regulating the observed ROS-mediated autophagic cell death, we acknowledge that the anti-tumor effects of a multi-component botanical extract like EEAL are likely multi-faceted. Targets such as EGFR may act as upstream regulators, while BCL2 and CASP3 may mediate cross-talk between autophagy and apoptosis. Therefore, the current study provides a focused mechanistic insight, and further investigation into these additional core targets will be essential to fully elucidate the complex pharmacological network of EEAL. Additionally, as EEAL contains 30 identified compounds, it remains unclear whether its anti-tumor efficacy arises from a single dominant molecule or synergistic interactions among multiple components. This multi-target complexity is inherent in traditional herbal medicines. Future research will focus on bioactivity-guided fractionation to isolate specific compounds and further elucidate their individual contributions to ROS-mediated autophagic cell death.

## 5 Conclusion

Our findings indicate that EEAL may exert its anticancer effects against colorectal cancer by inhibiting the AKT/mTOR/HIF-1 pathway, leading to oxidative stress and mitochondrial dysfunction, which in turn triggers excessive autophagy and compromises cell membrane integrity. Future research should focus on identifying the active components responsible for this effect while also conducting *in vivo* inflammatory studies to validate the reliability of our findings.

## Data Availability

The original contributions presented in the study are included in the article/supplementary material. Further inquiries can be directed to the corresponding authors.
